# A systematic review on the role of mitochondrial dysfunction/disorders in neurodevelopmental disorders and psychiatric/behavioral disorders

**DOI:** 10.3389/fpsyt.2024.1389093

**Published:** 2024-06-28

**Authors:** Daniela V. Pinto Payares, Logan Spooner, Jennifer Vosters, Samantha Dominguez, Lauren Patrick, Ann Harris, Shibani Kanungo

**Affiliations:** ^1^ Department of Student Affairs, Western Michigan University Homer Stryker MD School of Medicine, Kalamazoo, MI, United States; ^2^ Department of Medical Library, Western Michigan University Homer Stryker MD School of Medicine, Kalamazoo, MI, United States; ^3^ Department of Pediatric and Adolescent Medicine, Western Michigan University Homer Stryker MD School of Medicine, Kalamazoo, MI, United States; ^4^ Department of Medical Ethics, Humanities and Law, Western Michigan University Homer Stryker MD School of Medicine, Kalamazoo, MI, United States

**Keywords:** mitochondrial disorder, mitochondrial dysfunction, neurodevelopmental disorder, psychiatric disorder, ASD, ADHD, depression, anxiety

## Abstract

**Introduction:**

Mitochondrial diseases are known inborn errors affecting energy metabolism and are as common as chronic diseases such as diabetes, affecting approximately 1 in 5,000 people. The role of mitochondrial diseases/dysfunction has been highlighted in neurodevelopmental disorders like ASD, ADHD, intellectual disability, and speech delay, as well as various psychiatric conditions. Neurodevelopmental disorders are increasingly recognized as having behavioral and psychiatric symptoms. Our study aimed to investigate reports of mitochondrial disorders, noting neurodevelopmental disorders and psychiatric/behavioral conditions.

**Methods:**

This was done through a systematic review of literature from PubMed/MEDLINE, Scopus, and Cochrane Library up to November 2022.

**Results:**

We found 277 publications, of which 139 met the inclusion criteria. We mostly found review articles with mention of mitochondrial dysfunction/disorder in relation to ASD with brief mentions of psychiatric/behavioral comorbidities.

**Discussion:**

This suggests a need for broader research efforts beyond ASD to understand the relationship between mitochondrial disorder or dysfunction and various neurodevelopmental and psychiatric/behavioral comorbidities.

## 1 Introduction

Mitochondria are subcellular organelles with numerous functions, including energy production through oxidative phosphorylation (OXPHOS), calcium homeostasis, reactive oxygen species production, scavenging, regulation of apoptosis, and activating caspase family of proteases (1, 2). Defects in mitochondrial gene expression can lead to failure of respiratory chain enzymes and ATP synthase, impairing OXPHOS, which is essential for cellular energy production (3). Mitochondrial disorders are complex multisystemic diseases with heterogenous clinical manifestations and can affect every organ and system (2). Mitochondrial diseases are one of the most prevalent inborn errors in metabolism, affecting about every 1 in 5,000 people (4), up to 1 in 200 infants harbor mtDNA mutations (5). Primary mitochondrial disorders have been traditionally defined as resulting from any mutations in mitochondrial DNA (mtDNA) or nuclear DNA (nDNA). The complex relationship between nDNA and mtDNA genomes and more pathogenic variants (6) has generated debate on the definition based on OXPHOS function or mitochondrial structure and function (2). In primary mitochondrial disorders, high energy demand tissues are affected, resulting in neurologic, vision, hearing, neuromuscular, neurodevelopmental, and behavioral abnormalities.

Mitochondrial disorders are primarily of maternal inheritance but can also have autosomal dominant (AD), autosomal recessive (AR), and X-linked inheritance (6). Additionally, heteroplasmy can have varying effects on different family members (5).

Environmental stimuli producing reactive oxygen species (ROS) can affect mitochondrial function and, with stress, trigger energy deficits of a primary mitochondrial disorder (3). Mitochondrial dysfunction can affect DNA, protein, and lipids and has been implicated in type 2 diabetes, obesity, hypertension, and cardiovascular diseases, severe psychiatric neurological disorders, including schizophrenia, bipolar disorder, Alzheimer’s, and Rett Syndrome (7). Diagnosing mitochondrial disorders comes with many challenges: which tissue will provide the best sample, analyzing hundreds of mtDNA and nDNA gene variations, and epigenetic influences that are not fully understood.

### 1.1 Neurodevelopmental disorders

The Diagnostic and Statistical Manual of Mental Disorders (DSM)-5 defines neurodevelopmental disorders (NDDs) as conditions such as intellectual developmental disorder, communication disorders, autism spectrum disorder, attention deficit hyperactivity disorder, specific learning disorder, and motor disorders that affect developmental milestones since infancy, impairing personal, social, academic, motor, or occupational functions (8) as described below in **Table 1**.

**Table 1:** NDD characteristics per DSM-5.

Global developmental delay (GDD)	Impairment in all developmental domains (in children < 5 years of age).
Intellectual developmental disorder (intellectual disability, IDD/ID)	Affects both intellectual and adaptive functions such as problem-solving, planning, abstract thinking, communication, social participation, and academic functioning (in children > 5 years of age).
Communication disorders	Include language disorder, speech sound disorder, social communication disorder, childhood-onset fluency disorder, and stuttering affecting speech development.
Autism spectrum disorder (ASD)	Deficits in social communication and the presence of restricted, repetitive patterns of behavior or interests and can occur with or without ID.
Attention deficit hyperactivity disorder (ADHD)	Impairing levels of inattention, disorganization, and/or hyperactivity-impulsivity frequently overlaps with oppositional defiant disorder and often persists into adulthood.
Specific learning disorders	Learning deficits affecting specific academic skill with difficulties in reading, writing, and/or math.
Motor disorders	Include developmental coordination disorder, stereotypic movement disorder and tic disorders that interfere with social and academic performance (8). Cerebral palsy can be the severe end of a spectrum extending from minimal functional limitation to full dependency on care.

Earlier suggestions of an association between autism and disorders of mitochondrial oxidative phosphorylation (9–11), have moved towards environmental factors affecting mitochondrial dysfunction as a factor in the development and progression of ASD (7, 12–18). The mechanism of how mitochondrial dysfunction leads to an ASD phenotype is not known, but research suggests oxidative defects causing disruption in early brain development is a plausible mechanism (12).

### 1.2 Neurocognitive disorders

Neurocognitive disorders (NCDs) such as Alzheimer’s, Dementia, and Parkinsonism has acquired decline in cognitive functioning as prominent clinical feature. (8).

### 1.3 Behavioral/psychiatric disorders

Psychiatric disorders are behavioral, emotional, or cognitive dysfunctions in one or more areas, affecting social, occupational, and interpersonal functioning (19, 20). A few disorder related features are as described in **Table 2**:

**Table 2:** Behavioral/Psychiatric disorders characteristics per DSM-5.

Schizophrenia	Multiple signs and symptoms such as delusions, hallucination, disorganized speech, catatonic behavior, and apathy impairing occupational and/or social functioning.
Generalized anxiety disorder	Persistent and excessive worry in various contexts, such as work or school performance, with symptoms like restlessness, feeling on edge, fatigue, difficulty concentrating, muscle tension, and sleep disturbances.
Obsessive Compulsion Disorder	Obsessions (intrusive or recurrent and persistent thoughts, urges, or images) and/or compulsions (repetitive behaviors or mental acts performed in response to obsessions).
Depressive disorders	Depressed mood, there can be a loss of interest in activities, weight changes, sleep changes, psychomotor changes, fatigue, feelings of worthlessness or guilt, diminished ability to concentrate, and suicidal ideation.
Bipolar disorders	Episodes of hypomania and depression. Mania includes inflated self-esteem, decreased need for sleep, pressured speech, flight of ideas, distractibility, increase in goal-directed activity, and excessive involvement in activities impairing social or occupational function. Hypomania includes a shorter period (≥ 4 days) with expansive or elevated mood with the same manic symptoms as above and does not cause severe social or occupational impairment.
Cyclothymic disorder	At least 2 years of persistent hypomanic and depressive symptoms that do not meet the criteria for a full hypomanic or depressive episode.
Personality disorders	Enduring patterns of inner experience and behavior that deviate from an individual’s culture and must be pervasive and inflexible, have onset in early adulthood or earlier, and lead to distress or impairment.

### 1.4 Neurodevelopmental disorders and psychiatric/behavioral disorders

Studies have shown that NDD are increasingly recognized as having behavioral and psychiatric symptoms (21). ASD has been associated with higher rates of mood and anxiety disorders, obsessive-compulsive disorder, schizophrenia, and other psychiatric disorders. Suicide attempts are also more common in this population (21). A high comorbidity between autism and ADHD seem to be connected, presenting as social deficits from an etiological point of view (22).

Current literature highlights the role of mitochondrial disorder/dysfunction in numerous neurodevelopmental disorders, such as ASD, ADHD, intellectual disability, and speech delay (2, 4, 7, 11, 23). Mitochondrial abnormalities have been noted in various psychiatric disorders as well. Patients presenting with psychiatric symptoms later were found to have a primary mitochondrial disorder (24). Paranoid psychiatric symptoms and borderline personality disorder have been seen in patients later diagnosed with MELAS (25, 26). Individuals with hereditary sensorimotor neuropathy (HN) and mitochondrial mutations scored higher on depression screening scales and had a higher lifetime prevalence of psychiatric diagnoses and personality disorders (27).

Similarly, environmental factors affecting mitochondria, abnormalities in morphology and density of mitochondria, multiple mtDNA mutations, and SNPs have been implicated in the pathophysiology of many psychiatric disorders (28–30). While a genotype/psychiatric phenotype correlation has not been associated with any specific mitochondrial deletion or copy number variant (28), both animal and human subjects with mitochondrial disease presented with decreased neuronal function and had subsequent behavioral deficits consistent with psychiatric disorders (31). Mitochondrial dysfunction has been implicated in depression and schizophrenia. Specific mitochondrial disorders and dysfunctions have been identified in connection with certain psychiatric and neurodevelopmental disorders (23).

Our study aimed to investigate through a systematic review of literature reports of mitochondrial disorders noting neurodevelopmental disorders and psychiatric/behavioral disorders.

## 2 Methods

### 2.1 Search strategies, study selection, and data extraction

We conducted a (systematic) review of literature in PubMed/MEDLINE, Scopus, and Cochrane Library from inception to November 2022. The PubMed search strategy included keywords with asterisks (*) to capture the variation of the individual keyword as well as terms from the Medical Subject Headings Database (MeSH Database, https://www.ncbi.nlm.nih.gov/mesh/). Specific search tags used included[Mesh]: (“Mitochondrial Diseases”[Mesh] OR mitochondrial disease*) AND (“Neurodevelopmental Disorders”[Mesh] OR neurodevelopmental disorder*) AND (“Behavior”[Mesh] OR behavior*). Literature searches in Scopus and Cochrane Library included keywords (“mitochondrial disease*”) AND (“neurodevelopmental disorder*”) AND (behavior). We also searched the ClinicalTrials.gov database (https://clinicaltrials.gov/) using the keywords *“mitochondrial disease,” “neurodevelopmental disorder*, and *behavior* for any relevant clinical trials or studies. No filters or limiters were applied to ensure relevant literature was captured from inception to November 2022. All references identified from the searches were imported into Mendeley Reference Manager citation management software. Software and manual checks were made to remove duplicate citations. All remaining citations were imported and organized into a Microsoft Excel spreadsheet. Full texts of those citations were downloaded and imported into Mendeley.

Inclusion criteria:

- Full text
- Texts with English language availability
- Human subjects
- Translational cell studies with human cell lines

Exclusion criteria:

- No full text available
- Texts without English language available
- Animal studies

### 2.2 Classification of publications

Publications were categorized as animal if animals or animal-derived cells were the models used. Publications were classified as human studies when human subjects or cell lines were used. Human studies were further classified by type of publications as below:

- Review
- Metanalysis
- Cohort- Prospective/Retrospective
- Case Reports
- Case Series
- Case Control
- Observation
- Randomized control trial (RCT) including one translational study.
- *In Vitro* – included cell line studies
- Clinical Trials – all were listed in clinicaltrials.gov and/or Cochrane library

Publications were further noted if the paper discussed a mitochondrial disorder (mtDNA, nDNA, or OXPHOS defects) or a mitochondrial dysfunction and were screened for neurodevelopmental disorders and/or behavioral disorders were mentioned in them.

### 2.3 Data analysis

Data was examined to identify how many publications mentioned any neurodevelopmental disorder and/or psychiatric/behavioral disorder (Intellectual Disability: Mild, Intellectual Disability: Moderate, Intellectual Disability: Severe, Intellectual Disability (Nonspecific), Language Disorder, ASD, ADHD: Hyperactive, Cerebral Palsy, Schizophrenia, PTSD, Major Depressive Disorder: mild (general), Neurocognitive Disorder Possibly Due to Alzheimer, Bipolar disorder (general/nonspecific), Generalized Anxiety Disorder (general), Personality disorders (general/nonspecific), Neurocognitive Disorder Possibly Due to Parkinson’s, OCD, Dementia), in each type of publication (Review, Prospective, Meta, Cohort, Case Reports, Case Series, Case Control, Observation, Retrospective, RCT, *In Vitro*, Clinical Trials.). If any single publication mentioned more than one neurodevelopmental disorder and/or psychiatric/behavioral disorder, they were counted under each individual disorder category.

## 3 Results

**Figure 1:**
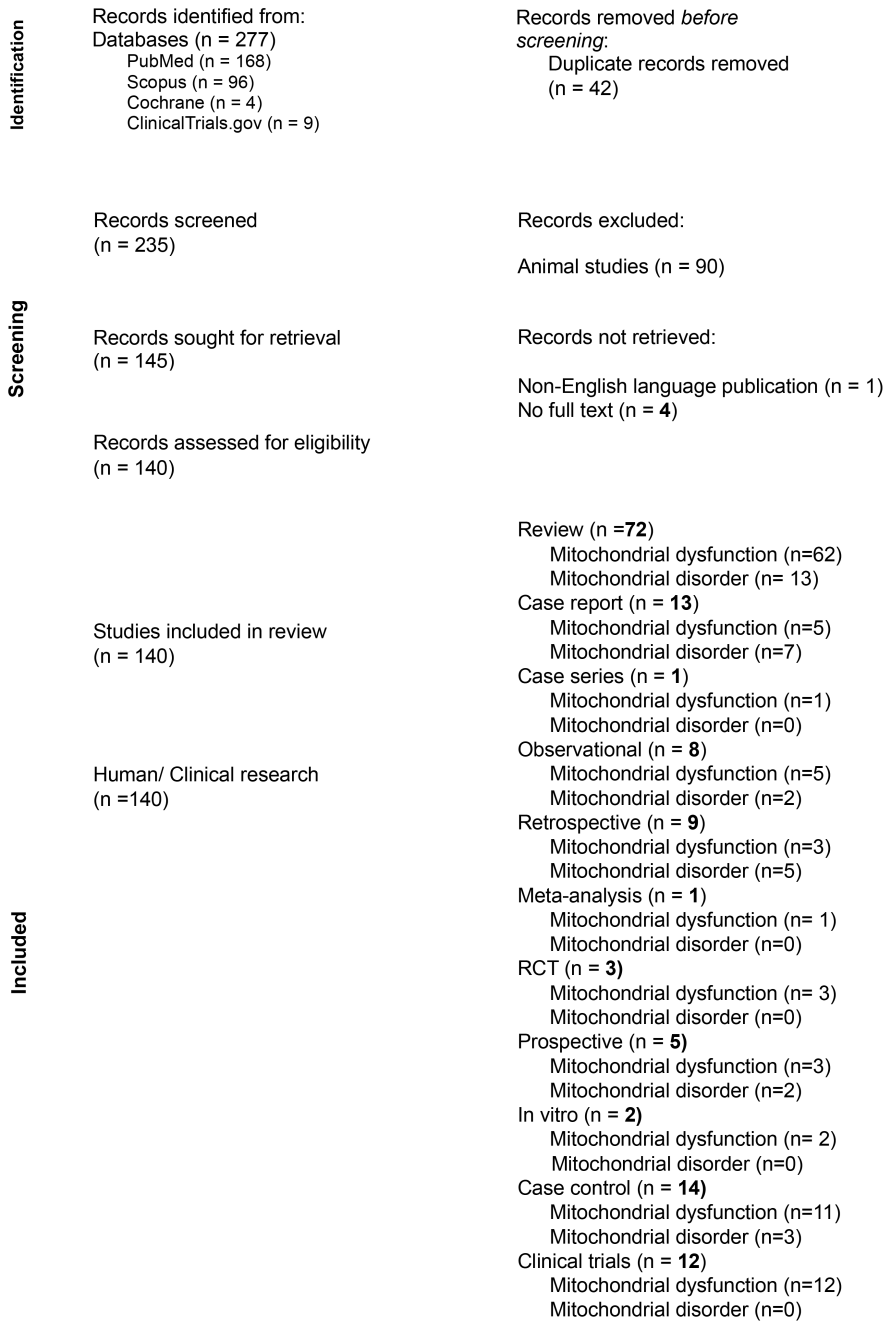
Identification and classification of publications from database searches.

Our literature search yielded 277 publications from the above four databases. PubMed returned 168 results, Scopus returned 96 results, ClinicalTrials.gov returned 9 results, and Cochrane Database returned 4 results. Of the 277 total publications, 42 duplicate records were removed. This led to 235 publications being screened with 95 meeting exclusion criteria and 139 meeting inclusion criteria articles. See **Figure 1** (flow chart) for more details.

Of the 139 publications included, 72 were identified as reviews where 62 noted mitochondrial dysfunction and 13 included mitochondrial disorders and or dysfunction; 13 were case reports with 5 discussing mitochondrial dysfunction and 7 relating to mitochondrial disorder. 1 case series noting mitochondrial dysfunction; 8 Observational with 5 reported on mitochondrial dysfunction and 2 on mitochondrial disorder; 5 prospective studies contained 3 mitochondrial dysfunction and 2 mitochondrial disorders; 9 retrospective publications were found, 3 of which was mitochondrial dysfunction-related and 5 mitochondrial disorders; 14 case-control studies 11 mentioned dysfunction of the mitochondria while 3 pertained to mitochondrial disorders; 3 randomized clinical trials were found with 3 discussing mitochondrial dysfunction and 0 relating to mitochondrial disorders; 2 *in vitro* studies both noting mitochondrial dysfunction; 1 meta-analysis mentioning mitochondrial dysfunction; 12 clinical trials where all 12 mentioned mitochondrial dysfunction.

**Figure 2:**
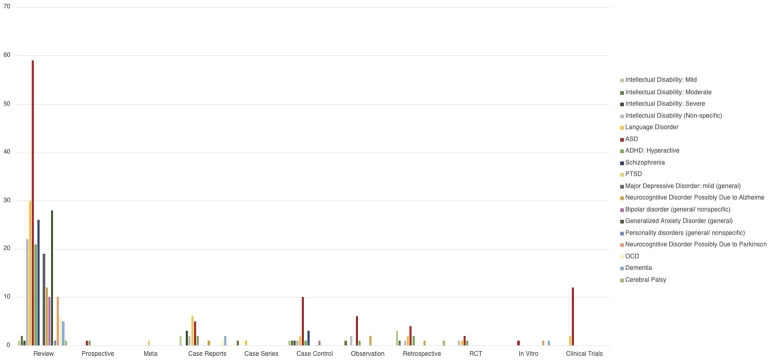
Distribution of the number of number of NDD and behavioral disorders in the different type of papers analyzed. The bars represent the number of paper the condition was mentioned in. Some papers mentioned more than one condition, and in those cases the paper were counted more than once.

Review of each type of publication with mention of any NDD, neurocognitive disorder and/or psychiatric/behavioral disorder (Intellectual Disability: Mild, Intellectual Disability: Moderate, Intellectual Disability: Severe, Intellectual Disability (Nonspecific), Language Disorder, ASD, ADHD: Hyperactive, Cerebral Palsy, Schizophrenia, PTSD, Major Depressive Disorder: mild (general), Neurocognitive Disorder Possibly Due to Alzheimer, Bipolar disorder (general/nonspecific), Generalized Anxiety Disorder (general), Personality disorders (general/nonspecific), Neurocognitive Disorder Possibly Due to Parkinson’s, OCD, Dementia) is shown in **Figure 2**.

Neurodevelopmental disorders mentioned in publications are detailed in **Table 3**. Neurocognitive disorders mentioned in publications are detailed in **Table 4** and Behavioral/psychiatric disorders mentioned in publications are detailed in **Table 5**.

**Table 3:** Neurodevelopmental disorders mentioned in the different types of publications.

Article Type	# of articles where NDD/Behavioral disorder were discussed								
	Intellectual Disability: Mild	Intellectual Disability: Moderate	Intellectual Disability: Severe	Intellectual Disability (Non-specific)	Language Disorder	ASD	ADHD: Hyperactive	Cerebral Palsy	TOTAL
**Review**	1	2	1	22	30	59	21	1	137
**Prospective**						1	1		2
**Meta**									0
**Case Reports**	2		3	2	6	5	2		20
**Case Series**		1			1				2
**Case Control**	1	1	1	1	2	10	1		17
**Observation**		1		2		6	1		10
**Retrospective**	3	1		1	2	4	2	1	14
**RCT**				1	1	2	1		5
**In Vitro**						1			1
**Clinical Trials**					2	12			14
**TOTAL**	7	6	5	29	44	100	29	2	222

**Table 4:** Neurocognitive disorders mentioned in the different types of publications.

Article Type	# of articles where NDD/Behavioral disorder were discussed			
	Neurocognitive Disorder Possibly Due to Alzheimer	Neurocognitive Disorder Possibly Due to Parkinson's	Dementia	TOTAL
**Review**	12	10	5	27
**Prospective**				0
**Meta**				0
**Case Reports**	1		2	3
**Case Series**				0
**Case Control**				0
**Observation**	2			2
**Retrospective**	1			1
**RCT**				0
**In Vitro**		1	1	2
**Clinical Trials**				0
**TOTAL**	16	11	8	35

**Table 5:** Behavioral/psych disorders mentioned in the different types of publications.

Article Type	# of articles where NDD/Behavioral disorder were discussed							
	Schizophrenia	PTSD	Major Depressive Disorder: mild (general)	Bipolar disorder (general/ nonspecific)	Generalized Anxiety Disorder (general)	Personality disorders (general/ nonspecific)	OCD	TOTAL
**Review**	26		19	10	28	1	6	90
**Prospective**								0
**Meta**		1						1
**Case Reports**							1	1
**Case Series**								0
**Case Control**	3			1				4
**Observation**								0
**Retrospective**								0
**RCT**								0
**In Vitro**								0
**Clinical Trials**								0
**TOTAL**	29	1	19	11	28	1	7	96

## 4 Discussion

We examined 139 publications on mitochondrial disease/dysfunction that highlighted the role of mitochondrial disease/dysfunction in NDDs and behavioral/psychiatric disorders. **Tables 3** and **5** bring to light the lack of new research studying the relationship between mitochondrial dysfunction and NDDs and psychiatric/behavioral disorders. Additionally, the majority of articles were focused on ASD, leading to the question of whether ASD has a stronger correlation to mitochondrial disease or if it has more research funding.

There is limited research exploring mitochondrial diseases, though more common than is currently understood, affecting 1 in 5000 people (4). Without strong clinical suspicion, mitochondrial diseases can be difficult to diagnose due to their heterogeneous presentation and multiorgan system involvement.

The contents of our systematic review search indicated mitochondrial dysfunction/disorder was mentioned in 100 articles investigating ASD. Of the NDDs and behavioral/psychiatric disorders we searched, the preponderance of the articles focused on elucidating the connection between ASD and mitochondrial function.

In a population-based study, 120 children with ASD were studied for potential etiologies. Of the 69 children tested, 11 likely had a mitochondrial etiology. This connection between mitochondrial function and ASD has led to a shift in terminology, classifying ASD based on pathophysiology—”idiopathic ASD” vs. “mitochondrial ASD” (12). Another study showed that up to 16% of individuals with mtDNA deletions, while only 3.3% of the healthy control had mtDNA deletions. The study concluded that mtDNA mutations were statistically more common in patients with ASD compared to controls (18).

As the relationship between ASD and mitochondrial dysfunction becomes clear, the question of pathophysiology must be accounted for. Individuals with ASD frequently have multiorgan comorbidities. These diverse disorders may be accounted for by concomitant mitochondrial dysfunction. If the mitochondria are affected in ASD and cannot produce enough ATP, it naturally follows that high-energy organ systems would be negatively impacted, resulting in comorbidities (32).

Despite the significant amount of research dedicated to ASD and mitochondrial dysfunction, there are gaps in our knowledge. While ASD is classified as an NDD, there is a behavioral component to its presentation, which is unexplored in these articles. In our review, we found the link between behavioral/psychiatric disorders and mitochondrial dysfunction to be vastly uncharted. There were only 131 mentions of all behavioral/psychiatric disorders, some of which are very brief. Conversely, ASD, which is extensively researched, was the main topic of 100 articles, questioning research bias for such correlation between ASD and mitochondrial disease,.

With an annual budget of over $40 billion, the National Institutes of Health (NIH) is the largest single public funder of biomedical and behavioral research in the world (33). Of the NDDs, neurocognitive disorders, and behavioral/psychiatric disorders we found in our systematic investigation, allocation of NIH research funds (from largest to least) were on: Alzheimer’s disease; intellectual and developmental disabilities; ASD; schizophrenia; Parkinson’s disease; anxiety disorders; MDD; bipolar disorder; ADHD, and Tourette’s.

NIH funding of ASD has increased from $118 million in 2008 to $306 million in 2022, while no funding was provided for mitochondrial disorders (34). This research has resulted in standardized screening tools, such as MCHAT-R/F, that allow pediatricians to identify ASD effectively and systematically. The prevalence of ASD is 1/36 (35) in comparison to 1/5000 for mitochondrial disease. Only with more research and improved standardized screening for mitochondrial disease will we truly know if this difference in prevalence is as substantial as it first appears.

In addition to ASD, our search produced 47 mentions of intellectual disability. One study found a link between genetic variation in mtDNA and ID. In this study, individuals with ID presented with motor coordination deficits, attention deficits, epilepsy, heart conditions, and GI disorders. 79% of the genetic variations were identified as SNPs (36). This study also linked mtDNA mutations to ASD, bipolar disorder, schizophrenia, MELAS, LHON, and Leigh Syndrome, among others. This indicates a common mitochondrial denominator between the NDDs and behavioral/psychiatric disorders we are analyzing in this paper.

Our search generated 44 references to language disorders. One article emphasized the importance of creatine in brain functions. Creatine formation involves mitochondria, and creatine is necessary in the expenditure of ATP. Therefore, in cases of mitochondrial disruption of the creatine pathway, individuals present with intellectual and language disorders (37).

This systematic review search yielded 29 mentions of ADHD. ADHD and ASD were found to have several concomitant biological components, including: increased oxidative stress, increased toxic metal burden, decreased methylation, mitochondrial dysfunction, and cerebral hypoperfusion. These shared features indicate mitochondrial dysfunction plays a key role in the development of both conditions (38). Another article investigated the role of parent-of-origin effects (POE) in the development of ADHD. Specifically, situations in which the unequal passing of genetic information affects mitochondrial function. However, no single mechanism of inheritance for ADHD has been identified, leading us to conclude mitochondrial dysfunction regulating the ADHD phenotype (39).

Similarly, in our analysis of behavioral and psychiatric disorders, the bioenergetic deficits seen in the aforementioned NDDs can contribute to the etiology of several psychiatric disorders, such as schizophrenia, MDD, and bipolar I disorder.

A cross-sectional study done with 12 children with mitochondrial disorders, 33% had depressive symptoms, and 50% of these participants had problems with anxiety (40). In a similar study, 36 adults with confirmed mitochondrial disorders found the lifetime prevalence of major depressive disorder is 54%, 17% for bipolar disorder, 11% for generalized anxiety disorder, and 11% for panic disorder. 17% of subjects were at current risk for suicide, and 8% were considered high risk (41). This raises a question about the correlation between mitochondrial function and prevalent psychiatric disorders.

Twenty-nine articles in our review mention schizophrenia and bring forth compelling evidence of mitochondrial involvement in its pathophysiology, the first of which is mitochondrial morphology. Studies have found mitochondrial swelling and hyperplasia in schizophrenic individuals, as well as inconsistent evidence of altered oxidative phosphorylation due to ETC complex dysfunction. Disease inheritance also implicates mitochondrial involvement, as the rates of schizophrenia are higher in female relatives. Studies indicate that the familial risk of developing schizophrenia was significantly higher in family members who shared mtDNA (42).

Similarly, bipolar disorder was only mentioned 11 times in our systematic review. In addition to several mtDNA SNPs found to correlate with bipolar disorder, previous studies have uncovered significant differences in mitochondrial size and distribution in the CNS of bipolar patients when compared with controls (42). Additionally, as evidenced by increased lipid peroxidation and antioxidant enzyme alterations, it is believed there is an overproduction of ROS in bipolar patients (42). A final, albeit inconsistent, finding points towards a shift from oxidative phosphorylation to glycolysis in bipolar individuals. While intriguing, these findings will remain inconclusive without proper investigation (42).

MDD, referenced 19 times in our literature search on mitochondrial disease, has a lifetime prevalence of 5-17%. Research found in all stress models of depression, there was a bioenergetic impairment such as decreased ATP, increased ROS, and/or decreased antioxidant defense. All of which correspond to an underlying mitochondrial dysfunction, making MDD another example of an exceptionally common condition with evidence of mitochondrial involvement that has not been suitably explored (43).

Delving into neurocognitive disorders, our search noted 16 papers connecting Alzheimer’s disease and mitochondrial dysfunction, even theorizing that deficits in mitochondrial function may be the primary event leading to various neurodegenerative disorders, including schizophrenia and Alzheimer’s disease. The leading postulation of Alzheimer’s pathogenesis involves increased oxidative stress due to mitochondrial impairment. One article suggested an accumulation of deletions and point mutations in mtDNA results in the progressive dementia seen in Alzheimer’s disease (44). Specifically, the most frequent deletion of 4977bp in mtDNA contributes to the reduced functionality of 1+ respiratory chain complexes, effectively blocking the ETC and increasing free radical generation. This increase in free radicals leads to more oxidative damage of mtDNA, continuing the cycle of mitochondrial dysfunction (44).

Our literature search disclosed 11 articles on Parkinson’s disease. One such article investigated the role of monoamine oxidases (MAOs) A and B may play in the pathophysiology of Parkinson’s disease. MAOs are critical to the proper functioning of synaptic neurotransmission, but the byproducts of the reaction have neurotoxic potential. This article suggests in cases where these enzymes are overactive, the accumulation of the byproducts could lead to mitochondrial damage and result in the Parkinsonian phenotype (45).

In conclusion, while these conditions may seem disparate in appearance or clinical presentation, they are all connected by an underlying etiology: mitochondrial dysfunction. ASD, due to its generous research funding, appears to have a stronger correlation with mitochondrial dysfunction. However, with the understanding of research bias, it is more likely that these other conditions have the same connection to mitochondrial impairment but lack the funds to fully investigate.

## 5 Limitations

Diagnosing mitochondrial disorders comes with many challenges: which tissue will provide the best sample, analyzing hundreds of mtDNA and nDNA gene variations, and epigenetic influences that are not fully understood. This makes mitochondrial disease difficult to study, as these difficulties indicate it may frequently go undiagnosed.

In addition to diagnostic complications, treatment and prognostic tools also present challenges. On average, people with mitochondrial disease have 16 major medical issues stemming from dysfunctional energy production (46). Even with research still in its early stages, over 350 pathogenic gene variants have been connected to mitochondrial disease (47). This makes a standardized test to detect mitochondrial disease exceptionally complicated.

With advanced diagnostics, novel therapeutics, and individualized research, we may find mitochondrial defects play a major role in all chronic conditions. That is the future required in order to properly identify and treat mitochondrial disease (48).

## 6 Future steps

Given the aforementioned limitations, there is much that requires improvement in order to enhance care of patients with mitochondrial disease. Specifically, more clinical trials and randomized control trials (RCT) are needed. Of the extensive search we conducted, only 12 were clinical trials and 3 were RCT. 72 of the 139 articles we examined were reviews. We need to cultivate interest, encourage new studies, and advance our understanding of mitochondrial disease.

## Data availability statement

The raw data supporting the conclusions of this article will be made available by the authors, without undue reservation.

## Author contributions

DP: Writing – review & editing, Writing – original draft. LS: Writing – review & editing, Writing – original draft. JV: Writing – review & editing, Writing – original draft. SD: Writing – review & editing, Writing – original draft. LP: Writing – review & editing, Writing – original draft. AH: Writing – review & editing, Writing – original draft. SK: Writing – review & editing, Writing – original draft.
